# Mechanism of *Trypanosoma cruzi* Placenta Invasion and Infection: The Use of Human Chorionic Villi Explants

**DOI:** 10.1155/2012/614820

**Published:** 2012-05-30

**Authors:** Ricardo E. Fretes, Ulrike Kemmerling

**Affiliations:** ^1^Department of Histology and Embryology, Faculty of Medicine, Universidad Nacional Córdoba, 5000 Cordoba, Argentina; ^2^IICSHUM and Cathedra of Histology, Embryology and Genetic, Health Department, Universidad Nacional La Rioja, 5300 La Rioja, Argentina; ^3^Program of Anatomy and Developmental Biology, Institute for Biomedical Sciences, Faculty of Medicine, University of Chile, 8380453 Santiago, Chile

## Abstract

Congenital Chagas disease, a neglected tropical disease, endemic in Latin America, is associated with premature labor and miscarriage. During vertical transmission the parasite *Trypanosoma cruzi* (*T. cruzi*) crosses the placental barrier. However, the exact mechanism of the placental infection remains unclear. We review the congenital transmission of *T. cruzi*, particularly the role of possible local placental factors that contribute to the vertical transmission of the parasite. Additionally, we analyze the different methods available for studying the congenital transmission of the parasite. In that context, the *ex vivo* infection with *T. cruzi* trypomastigotes of human placental chorionic villi constitutes an excellent tool for studying parasite infection strategies as well as possible local antiparasitic mechanisms.

## 1. Introduction

Chagas disease was first described by the Brazilian physician, Carlos Chagas, in 1909. He identified the causal agent, the protozoan *Trypanosoma cruzi *(*T. cruzi*), the vectorial transmission and insect reservoirs as well as the clinical signs and symptoms. In other words, he described the complete cycle of the disease and suggested the possibility of congenital transmission [1].

In the human villous hemochorial placenta, fetal and maternal tissues are separated by a fetal epithelium (the trophoblast). Within the villous placenta, a single multinucleated cell layer (syncytiotrophoblast) contacts maternal blood within the intervillous space. Beneath the syncytiotrophoblast reside replicating progenitors (cytotrophoblast) that are separated by a basal lamina from the connective tissue of villous stroma containing vascular endothelium, fibroblasts, and macrophages. The syncytiotrophoblast forms a surface of about 12 m^2^ that contacts maternal blood. Therefore, in case of women with Chagas disease, the parasite has the opportunity to interact with a large cellular surface.

The mechanism of *T. cruzi *congenital transmission can be studied in different ways. One of the possible ways is through the dual placental perfusion system. The dual perfusion that simulates the maternal and fetal circulation could be an excellent method to study the mechanism of the transmission of the parasite [2]. However, this method requires complex equipment and very experienced users. Another possibility is the *ex vivo* infection of human chorionic villi explants, in which samples of placental tissue (chorionic villi) can be challenged *in vitro* with *T. cruzi* or other pathogens. This system is preferred to analyze the process of infection of the placental barrier, although the immune system does not participate. The analysis of infection in animal models is not treated in this paper because their placentas are not similar to human placenta. Also, pathological findings of placentas are not deeply described because they represent the final stage of the placental infection process, and so, it is hard to understand an ongoing process.

The aim of the present paper is to review the congenital transmission of *T. cruzi*, particularly the role of possible local placental factors which contribute to the vertical transmission of the parasite and can be studied in the *ex vivo* infection model of human chorionic villi explants.

## 2. Chagas Disease

Chagas disease develops in three phases. The acute phase is first developed, immediately after infection, with high levels of parasitemia and symptoms in only some patients (regional lymph node enlargement, bipalpebral unilateral edema, or Romaña's sign, and characteristic electrocardiogram alterations). In most cases, acute infection is not accompanied by clinical findings, thus moving onto the latent phase that can last for months or years. The chronic phase, present in 30% of infected individuals, is associated with mega colon, mega esophagus, degeneration of the autonomous nervous system, arrhythmias, and abnormal growth of the heart with progressive insufficiency, with evident negative impact on the patient's health. In this phase the disease can be handicapping, and can either be the concurrent or the direct cause of death. The course of the disease depends on diverse factors: parasite load at the site of inoculation, both the parasite's genetic group and strain, whether it is an infection *de novo* or reinfection, the host's immunologic status, and the type of vector (triatomid) [3, 4].

## 3. Congenital Chagas Disease

Congenital *T. cruzi* infection is associated with premature labor, low birth weight, and stillbirths [5–7]. Serologic prevalence among pregnant women may reach 80%, and rates of congenital infection vary from 1–21% [8–11]. In Argentina the transmission rate is estimated between 2–12% [12]. In Chile, in two of the endemic regions (IV and V regions), the congenital transmission rate of the parasite is 8.4% [13]. According to WHO/PAHO the number of infected women at fertile age is approximately 1.8 million and it is estimated that 14,400 neonates are being infected each year [14], this is another reason why this form of transmission becomes epidemiologically more important. Additionally, congenital transmission is partly responsible for the “globalization of Chagas's disease” [10, 15].

Congenital Chagas transmission implies a *T. cruzi *seropositive mother and a postpartum detection of parasites in the newborn. Congenital Chagas disease is diagnosed by direct microscopic examination of blood samples, PCR, or standard serological assays. The latter can be carried out in infants when IgG antibodies transferred from the mother have been eliminated (8-9 months after birth).

During congenital transmission, the parasite reaches the fetus by crossing the placental barrier [16–22]. The fact that only a percentage of the infected mothers transmit parasites to their fetuses raises the question of the ability of the placenta as well as the immunological status of mother and fetus/newborn to impair the parasite transmission. Therefore, it is thought that congenital Chagas disease is the product of a complex interaction between the parasite, the maternal and fetus/newborn immune responses, and placental factors [9, 18].

## 4. The Parasite

*T. cruzi* is a haemoflagellated protozoan of the Kinetoplastida order and Trypanosomatidae family [22]. The parasite's biological cycle includes three cellular forms characterized by the relative positions of the flagellum, kinetoplast, and nucleus [23]: (1) trypomastigotes: approximately 20 *μ*m in length and sub terminal kinetoplast. They constitute the nonreplicative, mammalian infecting cellular form that is found in the blood and in the posterior intestine of triatomids. In mammals, this is the cellular form that disseminates infection through blood. (2) Epimastigotes: also 20 *μ*m in length with a kinetoplast anterior to the nucleus. They represent the multiplying parasite form in the triatomid intestine. (3) Amastigotes: approximately 2 *μ*m in diameter, rounded, with no emergent flagellum. It multiplies within the mammalian host cells, forming nests, until they rupture after several cell divisions. Before their release from the host cells, amastigotes differentiate into trypomastigotes which once released, invade the blood stream; they may then enter any other nucleated cell. Epimastigotes can be grown in axenic cultures while amastigotes are grown in cultured mammalian cells, releasing trypomastigotes that can be harvested to perform *in vitro* assays.

*T. cruzi *displays great biological, biochemical, and genetic diversity; therefore different strains of the parasite have been identified and classified into six discrete typing units (DTUs) [24, 25]. Strains of *T. cruzi* have been involved in different clinical forms of Chagas disease [26, 27], thus implicating a different genetic population in tissue tropism, replication, and virulence, and in consequence in disease outcome. *T. cruzi* strains corresponding to different DTUs might have relevant consequences on congenital transmission and fetal/neonatal pathology, even though Virreira et al. [28] and Burgos et al. [9] concluded that congenital transmission of *T. cruzi* is not associated with genetic polymorphism of *T. cruzi*. However, Solana et al. [29] and Triquell et al. [21] described biological differences among subpopulations of *T. cruzi* in experimental vertical transmission and placental infection.

## 5. Mother and Fetus/Newborn Immune Response

The immune system is fundamental to protect the mother against the environment, and to prevent damage to the fetus. During pregnancy the maternal immune system is characterized by a reinforced network of cellular and molecular recognition, communication, trafficking and repair; it raises the alarm to maintain the wellbeing of the mother and the fetus. On the other hand, the fetus provides a developing active immune system that will modify the way the mother responds to the environment, providing a uniqueness of the immune system responses during pregnancy [30]. A crucial factor to stop, limit, or permit the development of fetal/neonatal infection relates to the capacity of the mother and fetus/newborn to mount innate and/or specific immune response(s) against pathogens. Clinical studies have shown a strong association between intrauterine infections and pregnancy disorders such as abortion, preterm labor, intrauterine growth retardation, and preeclampsia [31]. As described above, congenital *T. cruzi* infection is associated with some of these pathologies [5–7, 15]. Production of proinflammatory cytokines can be observed in uninfected newborn from infected mothers [15]. Contrarily, the levels of inflammation markers and activation of NK cells are rather low in congenitally infected newborns [32]. This data highly suggests a protective role of such innate defenses in an uninfected newborn from infected mothers. On the other hand, maternal *T. cruzi*-specific IgG antibodies play protective roles in mothers and in fetuses when antibodies are transferred through the placenta [33] and also may contribute to a reduction in parasitaemia [15].

## 6. Placenta

**Figure 1:**
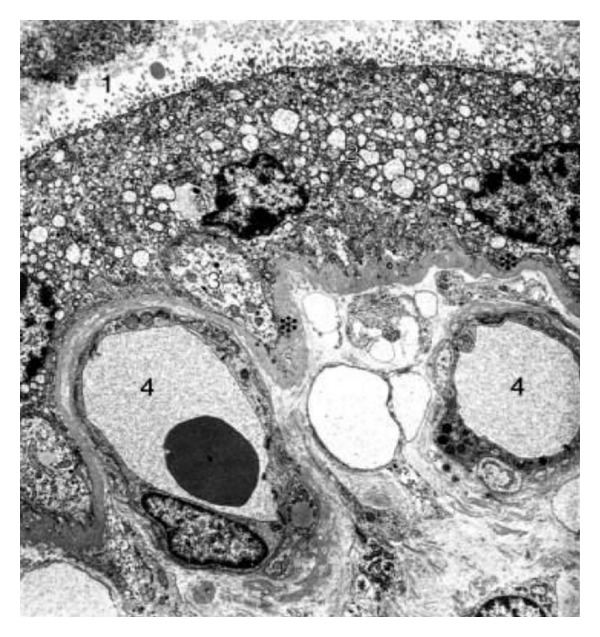
Electron micrograph of a chorionic villous human placenta. Picture depicts the intervillous space (1) and the placental barrier formed by the syncytiotrophoblast (2), a discontinuous cytotrophoblast (3), basal laminae (asterix), conective tissue, and fetal vessels (4).

The placenta is the principal site for the exchange of nutrients and gases between the mother and fetus. This organ plays an important role in hormone, peptide, and steroid synthesis necessary for a successful pregnancy [34]. The human placenta is classified as a hemochorial villous placenta in which the free chorionic villi, formed by the trophoblast and the villous stroma, are the functional units. The trophoblast contacts maternal blood in the intervillous space, and it is separated by a basal lamina from the villous stroma, which is connective tissue containing the vascular endothelium, fibroblasts, and macrophages (Figure 1) [35]. Trophoblast, basal lamina, and villous stroma with the endothelium of fetal capillaries form the placental barrier that must be crossed by different pathogens, including *T. cruzi*, to infect the fetus during vertical transmission [16–22, 36–41].

Placentas from mother with acute Chagas disease (high parasitaemia) show severe histopathological changes, such as extensive necrosis, inflammatory infiltrate, and amastigote nests [5]. Contrarily, placentas from mother with chronic Chagas disease do not present necrotic foci and inflammatory infiltrate. Although parasite antigens can be visualized in the villous stroma, the typical amastigote nests are not present [22]. In accordance with these results, in *ex vivo *infected placental explants, though parasite antigens and DNA can be detected [17, 18, 42], amastigote nests are not observed. Only few individual parasites can be detected. This evidence suggests that antiparasite mechanisms may exist in the placental tissue of women suffering chronic Chagas disease.

## 7. Possible Antiparasitic Mechanisms of the Placenta

We updated the importance of the presence of the causal agent of Chagas disease in the intervillous space of human placentas, the viability of the parasite in this environment, and the process of infection of the placental tissue, mainly by *ex vivo* and *in vitro* studies.

**Figure 2:**
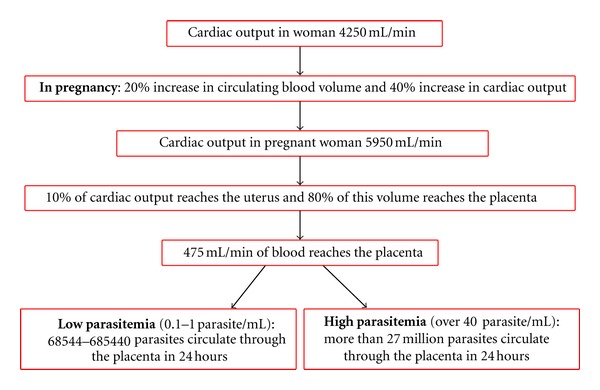
Estimation of *T. cruzi *contact with the placenta in infected mothers.

1. Clearance of *T. cruzi* from the intervillous space is associated with the risk of congenital transmission. Thus, a high parasitaemia, as in acute infection, correlates with a higher transmission rate [15, 18, 43, 44]. Thus the amount of parasites could be an important risk factor for mother to fetus transmission of *T. cruzi*. There are only few publications analyzing the survival of *T. cruzi* in the placental environment. Triquell et al. [21], employing chorionic villi *ex vivo* and *in vitro* infection model cocultured with trypomastigotes from two different strains of *T. cruzi*, observed that one of the strains presents a better survival rate than the other in the placental environment. Furthermore, the two strains of *T. cruzi* respond differently when they were treated with placental media. Therefore, the great biological, biochemical, and genetic diversity of the parasite may determine, at least partially, the capacity of placental infection. These results open a new concept, that placenta might exert a clearance of the parasite from the intervillous space, and that different populations of *T. cruzi* have different survival capacities in that environment.
2. Contact time between *T. cruzi* and the trophoblast in the intervillous space: the time that the parasite remains in the intervillous space in contact with the syncytiotrophoblast is poorly known. Placental barrier is constituted by the trophoblast tissue, that comprises a continuous multinucleated, nonreplicating cell layer, the syncytiotrophoblast, a replicating layer of cytotrophoblasts that fuses with the STB, a basal lamina, and an underlying villous stroma or connective tissue, that includes vascular endothelium [35]. The placental barrier must be crossed by the parasites, therefore the time that *T. cruzi* trypomastigotes stay in the intervillous space and interact with the syncytiotrophoblast is of outmost importance. Shippey et al. [45] in a dual perfusion system of placental cotyledons observed *T. cruzi* DNA in the maternal effluents at 30 min, 60 min, and 90 min after injection an only one bolus of *T. cruzi *trypomastigotes through the maternal perfusate. There was no parasite DNA in the fetal effluent, indicating there was no passage to fetal circulation despite the great concentration of parasites injected. However, the perfusion time was only 120 min in these experiments. Contrarily, in the *ex vivo* infection of chorionic villi explants, a reproducible infection is obtained after 24 hours of coincubation with the parasite [17]. However, the perfusion experiments indicate that *T. cruzi* is present in the intervillous space at least for an hour and half. Despite this long time of interaction, *T. cruzi* was not able to invade or survive in the placental barrier, indicating a defense mechanism of the placental barrier against the causal parasite of Chagas.
3. Placental infection: the *ex vivo* and *in vitro* infection of human chorionic villi explants from human term placenta with the parasite is an excellent and easy way to study the mechanism of cellular and tissue invasion mechanisms. The explants can be kept in culture for several days [46], where constituent cells and tissues are in a more physiological condition than their isolated counterparts in monolayer cell culture models. Another advantage of this model is that the cells retain physical contact with the basal lamina and continue to receive paracrine growth factor signals from the underlying villous stroma. In our laboratories, we have established the optimal conditions for the *ex vivo* infection of chorionic villi explants with *T. cruzi* [17, 18, 20, 21, 36–41, 47]. The coincubation of 10^5^ or 10^6^ trypomastigotes produces a reproducible infection of the chorionic villi [17]. This parasite's concentration may seem to be extremely high, but if we consider the amount of blood that circulates through the placenta every day, and then calculate the number of parasites that reaches the placenta, the parasite concentration recommended for *ex vivo* infection is not high. Therefore, if we consider that the cardiac output in women is 4250 mL/min, and that during pregnancy the circulating blood volume increases in 20% and the cardiac output in 40%. Then the cardiac output in pregnant women is 5950 mL/min. From this output, 10% reaches the pregnant uterus and 80% of this volume reaches the placenta. Taking into account all the data, a volume of 475 mL/minute of blood reaches the placenta [48]. Considering a parasitemia as low as 0,1 to 1 parasite/mL, a total of 68544 to 685440 parasites circulate through the placenta in 24 hours (Figure 2). On the other hand, in pregnant women with acute Chagas disease, Torrico et al. [11] have reported parasitemias over 40 parasites/mL; therefore, in this condition a total of 27 million parasites circulate in 24 hours in the placenta. If we consider all these data, our experimental conditions are not far from *in vivo* conditions.

The trophoblast, the first tissue that is in contact with the parasite in the intervillous space, constitutes a potential barrier to *T. cruzi*. We observed that the most notable tissue damage induced by *T. cruzi* in the chorionic villi explants is the trophoblast detachment and destruction. Additionally, the parasite induces selective disorganization of basal lamina, collagen I destruction [17], and apoptosis (especially in the trophoblast) in infected chorionic villi explants [49]. In accordance, we detected similar histopathological changes in placentas from women with chronic Chagas disease ([22] manuscript in this number of *J*. of Tropical Medicine). Therefore, the similar histopathological changes observed in chagasic mothers and in *ex vivo* infected chorionic villi, validates the latter model. The extracellular matrix alteration produced by *T. cruzi* not only promotes its motility in tissues and its entrance into cells, but also alters the presence of cytokines and chemokines, which in turn permits *T. cruzi* to modulate and evade both the inflammatory and immune responses [16, 50, 51]. Alternatively, these changes in ECM function may be part of a local placental defense mechanism, which could explain why only very few parasites can be detected in the placenta. Similar effects can be observed in the chorionic villi explants during *ex vivo* infection, since placental explants do not allow a sustained infection by *T. cruzi* [19]. Thus, the placenta controls the productive infection of *T. cruzi* in chorionic villous and exerts a protective function to fetus.

In order to understand the mechanism by which *T. cruzi* fuses with trophoblast plasma membrane, Calderón and Fabro [52] studied the interaction between syncytial plasma membranes from the human placenta and from the parasites, founding modifications of membrane lipids and proteins of the syncytiotrophoblast. Additionally, modifications of enzyme activities in the chorionic villi have been described [37–39]. For instance, placental alkaline phosphatase (PLAP) is a glycosylphosphatidylinositol anchored plasma membrane protein present in the trophoblast that decreases its activity in chagasic women and is related to congenital transmission [53]. In *ex vivo* infected chorionic villi, pretreatment of the placental tissue with phospholipase C prevents the parasite-induced decrease of PLAP activity and significantly reduces the infectivity of *T. cruzi*. These results are consistent with a pathogenetic role for placental alkaline phosphatase in congenital Chagas disease [36, 54]. Additionally, *T. cruzi* induces in the *ex vivo* infection model an increase of lysosomal vesicles in the trophoblast, which are fundamental during cell invasion of the parasite [36–39].

Analyzing the process of trophoblast infection by *T. cruzi* in an *in vitro* system culturing monolayer trophoblasts cells in interaction with infective trypomastigotes, it was shown that two types of chorionic villi trophoblasts, syncytiotrophoblast, and cytotrophoblast have a differential susceptibility to infection by the causal agent of congenital Chagas disease [55]. The reduced infection in the syncytiotrophoblast was associated to fewer viable parasites in the culture medium and increased levels of nitric oxide. These results emphasize the importance of the integrity of the first placental barrier, the syncytiotrophoblast, in order to avoid a *T. cruzi* infection of the inner trophoblasts or stromal chorionic villi cells. As it was described above, structural trophoblast alteration is a common sign of miscarriages and premature births in placentas of chagasic women and strongly associated to the congenital transmission of *T. cruzi*. In these clinical situations, the detachment of the first placental barrier is a common sign which is also associated to parasitism of the placental tissue [9]. Thus, differential infection between the first placental barrier with respect to the inner trophoblast or stromal cells could represent a mechanism of invasion of the human placenta by *T. cruzi*.

## 8. Conclusion

Congenital Chagas transmission constitutes an increasing public health problem, and it is responsible for the urbanization and spreading of the disease to nonendemic areas of Latin America, United States of America, and Europe [18, 21]. The fact that the *ex vivo* infection of the chorionic villi explants with the parasite reproduces the *in vivo* infection in terms of cellular changes and infectivity makes it an excellent tool for studying parasite infection strategies as well as possible local antiparasitic mechanisms.
